# Supplementary figures and images for: Nitrate and nitrite exposure leads to mild anxiogenic-like behavior and alters brain metabolomic profile in zebrafish
Source: PLoS One. 2020 Dec 31;15(12):e0240070. doi: 10.1371/journal.pone.0240070 (PMC7774831; doi:10.1371/journal.pone.0240070)

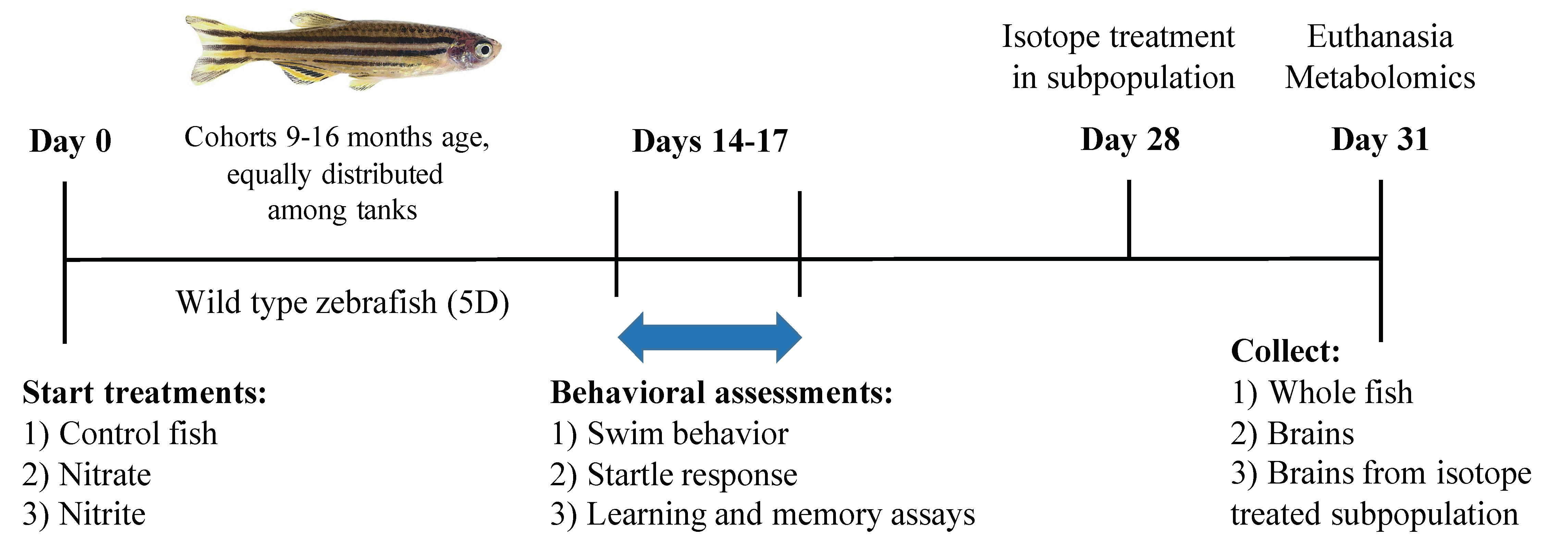

Supplement: S1 Fig — (TIFF) [file pone.0240070.s001.tiff]
